# Eleven years’ data of grassland management in Germany

**DOI:** 10.3897/BDJ.7.e36387

**Published:** 2019-09-27

**Authors:** Juliane Vogt, Valentin H. Klaus, Steffen Both, Cornelia Fürstenau, Sonja Gockel, Martin M. Gossner, Johannes Heinze, Andreas Hemp, Nobert Hölzel, Kirsten Jung, Till Kleinebecker, Ralf Lauterbach, Katrin Lorenzen, Andreas Ostrowski, Niclas Otto, Daniel Prati, Swen Renner, Uta Schumacher, Sebastian Seibold, Nadja Simons, Iris Steitz, Miriam Teuscher, Jan Thiele, Sandra Weithmann, Konstans Wells, Kerstin Wiesner, Manfred Ayasse, Nico Blüthgen, Markus Fischer, Wolfgang W. Weisser

**Affiliations:** 1 Technische Universität München, Terrestrial Ecology Research Group, School of Life Sciences Weihenstephan, Freising, Germany Technische Universität München, Terrestrial Ecology Research Group, School of Life Sciences Weihenstephan Freising Germany; 2 Westfälische Wilhelms-Universität, Institute of Landscape Ecology, Münster, Germany Westfälische Wilhelms-Universität, Institute of Landscape Ecology Münster Germany; 3 ETH Zürich, Institute of Agricultural Sciences, Zürich, Switzerland ETH Zürich, Institute of Agricultural Sciences Zürich Switzerland; 4 Technische Universität München, Terrestrial Ecology Research Group, School of Life Sciences Weihenstephan, Fresing, Germany Technische Universität München, Terrestrial Ecology Research Group, School of Life Sciences Weihenstephan Fresing Germany; 5 Martin-Luther-Universität Halle-Wittenberg, Institut für Agrar- und Ernährungswissenschaften, Halle, Germany Martin-Luther-Universität Halle-Wittenberg, Institut für Agrar- und Ernährungswissenschaften Halle Germany; 6 Friedrich Schiller Universität Jena, Institute for Computer Science, Heinz Nixdorf Chair for Distributed Information Systems, Jena, Germany Friedrich Schiller Universität Jena, Institute for Computer Science, Heinz Nixdorf Chair for Distributed Information Systems Jena Germany; 7 Friedrich Schiller Universität Jena, Institute of Ecology, Jena, Germany Friedrich Schiller Universität Jena, Institute of Ecology Jena Germany; 8 ThüringenForst, Forstliches Forschungs- und Kompetenzzentrum Gotha, Gotha, Germany ThüringenForst, Forstliches Forschungs- und Kompetenzzentrum Gotha Gotha Germany; 9 Swiss Federal Research Institute WSL, Forest Entomology, Birmensdorf, Switzerland Swiss Federal Research Institute WSL, Forest Entomology Birmensdorf Switzerland; 10 Universität Potsdam, Biodiversity Research/Systematic Botany, Institute of Biochemistry and Biology, Potsdam, Germany Universität Potsdam, Biodiversity Research/Systematic Botany, Institute of Biochemistry and Biology Potsdam Germany; 11 University of Bayreuth, Department of Plant Systematics, Bayreuth, Germany University of Bayreuth, Department of Plant Systematics Bayreuth Germany; 12 University of Ulm, Institute of Evolutionary Ecology, Ulm, Germany University of Ulm, Institute of Evolutionary Ecology Ulm Germany; 13 Westfälische Wilhelms-Universität, nstitute of Landscape Ecology, Münster, Germany Westfälische Wilhelms-Universität, nstitute of Landscape Ecology Münster Germany; 14 Justus-Liebig-Universität Gießen, Institute of Landscape Ecology and Resource Management, Gießen, Germany Justus-Liebig-Universität Gießen, Institute of Landscape Ecology and Resource Management Gießen Germany; 15 University of Bern, Institute of Plant Science, Department of Biology, Bern, Switzerland University of Bern, Institute of Plant Science, Department of Biology Bern Switzerland; 16 University of Natural Resources and Life Sciences BOKU, Institute of Zoology, Vienna, Austria University of Natural Resources and Life Sciences BOKU, Institute of Zoology Vienna Austria; 17 Senckenberg Gesellschaft für Naturforschung, Biodiversity and Climate Research Centre BiK-F, Frankfurt, Germany Senckenberg Gesellschaft für Naturforschung, Biodiversity and Climate Research Centre BiK-F Frankfurt Germany; 18 University Darmstadt, Ecological Networks, Darmstadt, Germany University Darmstadt, Ecological Networks Darmstadt Germany; 19 Johann Heinrich von Thünen Institute for Biodiversity, Braunschweig, Germany Johann Heinrich von Thünen Institute for Biodiversity Braunschweig Germany; 20 The University of Adelaide, Department of Biosciences, Adelaide, Australia The University of Adelaide, Department of Biosciences Adelaide Australia; 21 University of Ulm, Institute of Evolutionary Ecology, Ulm, Georgia University of Ulm, Institute of Evolutionary Ecology Ulm Georgia; 22 Universität Bern, Institute of Plant Science, Department of Biology, Bern, Germany Universität Bern, Institute of Plant Science, Department of Biology Bern Germany

**Keywords:** Grassland management survey, fertilisation, grazing, mowing, livestock units, Biodiversity-Exploratories, questionnaire, farming practice, grassland maintenance, nitrogen, temporal variation, intensification of grassland use

## Abstract

**Background:**

The 150 grassland plots were located in three study regions in Germany, 50 in each region. The dataset describes the yearly grassland management for each grassland plot using 116 variables.

General information includes plot identifier, study region and survey year. Additionally, grassland plot characteristics describe the presence and starting year of drainage and whether arable farming had taken place 25 years before our assessment, i.e. between 1981 and 2006. In each year, the size of the management unit is given which, in some cases, changed slightly across years.

Mowing, grazing and fertilisation were systematically surveyed:

***Mowing*** is characterised by mowing frequency (i.e. number of cuts per year), dates of cutting and different technical variables, such as type of machine used or usage of conditioner.

For ***grazing***, the livestock species and age (e.g. cattle, horse, sheep), the number of animals, stocking density per hectare and total duration of grazing were recorded. As a derived variable, the mean grazing intensity was then calculated by multiplying the livestock units with the duration of grazing per hectare [LSU days/ha]. Different grazing periods during a year, partly involving different herds, were summed up to an annual grazing intensity for each grassland.

For ***fertilisation***, information on the type and amount of different types of fertilisers was recorded separately for mineral and organic fertilisers, such as solid farmland manure, slurry and mash from a bioethanol factory. Our fertilisation measures neglect dung dropped by livestock during grazing. For each type of fertiliser, we calculated its total nitrogen content, derived from chemical analyses by the producer or agricultural guidelines (Table 3).

All three management types, mowing, fertilisation and grazing, were used to calculate a combined land use intensity index (LUI) which is frequently used to define a measure for the land use intensity. Here, fertilisation is expressed as total nitrogen per hectare [kg N/ha], but does not consider potassium and phosphorus.

Information on additional management practices in grasslands was also recorded including levelling, to tear-up matted grass covers, rolling, to remove surface irregularities, seed addition, to close gaps in the sward.

**New information:**

Investigating the relationship between human land use and biodiversity is important to understand if and how humans affect it through the way they manage the land and to develop sustainable land use strategies. Quantifying land use (the ‘X’ in such graphs) can be difficult as humans manage land using a multitude of actions, all of which may affect biodiversity, yet most studies use rather simple measures of land use, for example, by creating land use categories such as conventional vs. organic agriculture. Here, we provide detailed data on grassland management to allow for detailed analyses and the development of land use theory. The raw data have already been used for > 100 papers on the effect of management on biodiversity (e.g. Manning et al. 2015).

## Introduction

Grasslands can harbour high biodiversity and fulfil important ecosystem functions and services, such as food and habitat provision for livestock, protection of soil and water resources, carbon sequestration and aesthetic appeal (Carlier et al. 2009, Hönigová et al. 2012, Gossner et al. 2016, Simons et al. 2017). In addition to the conversion of grasslands to other land use forms, grasslands worldwide are also changed by land use intensification. Land use intensification of grasslands includes, for example, increased fertiliser input, application of pesticides, increased number of cuts in meadows or increased stocking densities in pastures (Humbert et al. 2009, Boch et al. 2016, Klaus et al. 2018. As a result of continued land use intensification, high value natural grasslands, i.e. extensively managed grasslands, have seen a decline throughout Europe (Veen et al. 2009).

Increasing management intensity in grasslands has been shown to decrease alpha, (i.e. local, diversity) and also beta diversity, i.e. intensification leads to homogenisation of communities across trophic groups including plant, invertebrates and birds (Humbert et al. 2009, Gossner et al. 2016, Manning et al. 2015, Renner et al. 2014, Socher et al. 2013). Intensification affects biodiversity directly and indirectly. For example, mowing itself and the use of conditioners, i.e. a farm implement that uses mechanical force to promote faster and more even drying of biomass, cause direct mortality of insects (Humbert et al. 2010a, Humbert et al. 2010b). Indirect effects include changes in plant community composition, for example, by increased fertilisation, that can then affect insect diversity.

Until now, little attention has been paid to long-term in-depth assessments of land use practices in grassland systems. The intensity and timing of mowing, grazing and fertilisation can differ within and between years on particular grasslands (Kleinebecker et al. 2018) and the effect of such variability on biodiversity changes is considerable (but see, e.g. Allan et al. 2014). Grassland management consists of various management components such as mowing, grazing or fertilisation that may jointly or singly affect biodiversity. Moreover, there are interactions between different management activities, for example, fertiliser application results in higher biomass production, which is often associated with more frequent mowing (Blüthgen et al. 2012, Busch et al. 2018, [Bibr B5226452][Bibr B5226327]). To understand more mechanistically how land use intensification in grasslands affects biodiversity, detailed information on grassland management is needed, ideally for a large number of grasslands over several years.

**Figure 1.**
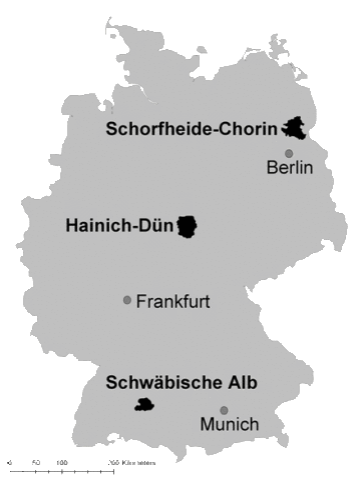
The three model regions of the *Biodiversity Exploratories* project in Germany.

Within the framework of the *Biodiversity-Exploratories* programme (www.biodiversity-exploratories.de), we have thoroughly monitored land use of 150 grassland plots for 11 years to investigate temporal variation in land management within three study regions in Germany (Fig. 1). These plots represented gradients of land use intensity typical for our study regions and were managed by mowing, grazing and fertilisation (Fischer et al. 2010). Detailed information on grassland management of all 150 grassland plots was obtained annually from farmers using a standardised questionnaire (Table 1). Here, we present the data of the corresponding management questionnaire that form the basis of most analyses of effects of land use intensification on biodiversity and ecosystem functioning in grasslands within the Biodiversity-Exploratories. With this dataset, we provide knowledge on how land use intensity in temperate grasslands varies across spatial and temporal scales. The components reported here also form the basis on an integrated land use intensity index used in the programme to study its integral effects on biodiversity in grasslands (Blüthgen et al. 2012).

## General description

### Purpose

The present dataset summaries management information collected from 2006 to 2016 for 150 grassland plots in three different regions of Germany. Data are based on annual interviews with the respective farmers, land owners or tenants involved in land management activity, using a standardised questionnaire.

## Project description

### Title

The Biodiversity Exploratories - functional biodiversity research

### Personnel

*Members of the steering committee of the BE*: Markus Fischer, Wolfgang Weisser, Manfred Ayasse, Christian Ammer, Nico Blüthgen, Ellen Kandeler, Birgitta König-Ries, Marion Schrumpf.

Within the infrastructure programme of the BE, local management teams in each region ensure the maintenance of survey plots and communication between scientists and local stakeholders. Furthermore, the grassland expert (technician) and the local manager (scientist) of each team are responsible for obtaining the information from the land user by carrying out the annual questionnaire, as well as including additional information by their own observations of the grasslands.

### Study area description

The biodiversity studies are carried out in 150 grassland plots managed in different intensities.

The grassland sites are distributed in three different regions within Germany including i) the Biosphere Reserve Schorfheide-Chorin ii) the Hainich-Dün Area and iii) the Biosphere-Area Schwäbische Alb.

The Schorfheide Chorin Exploratory site is situated in the North-East of Germany with an extent of approx. 1300 km². The geology is characterised by young glacial landscape of altitudes between 3-140 m a.s.l. with different soil types such as brown earth, lessivé, pararendzina, podzols and bog soils, resulting in diverse vegetation. The annual mean temperature is 8-8.5°C and the annual mean precipitation 500-600 mm.

The Hainich-Dün Exploratory site (approx. 1300 km²) in Central Germany consists of silty, loamy and clayey soil textures of the calcareous bedrock in altitudes between 285- 550 m a.s.l. The annual mean temperature is 6.5-8°C and the annual mean precipitation 500-800 mm.

The Exploratory Schwäbische Alb site (approx. 422 km²) in South West Germany consists of calcareous bedrock with karst phenomena in altitudes between 460-860 m a.s.l. with annual mean temperature of 6-7°C and mean precipitation of 700-1000 mm.

### Design description

For an advanced biodiversity research, three large-scale and long-term research sites were established in Germany serving as open research platforms for biodiversity and ecosystem research groups. The BE sustained the scientific infrastructure to develop the intellectual framework needed to address critical questions about changes in biodiversity and to evaluate the impacts of those changes for ecosystem processes.

The objectives of the BE are to understand i) the relationship between biodiversity of different taxa and levels, ii) the role of land use and management for biodiversity, iii) the role of biodiversity for ecosystem processes.

### Funding

The Biodiversity Exploratories are a German Science Foundation funded research project (DFG Priority Programme 1374).

## Sampling methods

### Study extent

We monitored 150 grassland plots across three regions in Germany for 11 years since 2006.

### Sampling description

Interviews with the land users took place retrospectively for the previous year on all permanently established 150 grassland sites since 2006, based on a standardised questionnaire, which was identical for all three exploratory regions.

We did not collect any organisms. During the interviews, the land users provided us with information according to their grassland management.

Linear mixed-effect models with logarithmically transformed response variables were calculated to detect temporal trends as well as differences between the exploratory regions (procedure lmer, implemented in R).

Land use intensity in the grasslands of our study regions ranged from low-intensive management, for example, meadows with only one cut per year and no fertilisation, to intensive management with four cuts per year and occasionally up to 400 kg N added per year and hectare. Very intensively-used grasslands which, in Central Europe, are characterised by up to seven cuts per year and regular fertilisation of about 400 kg/ha/yr nitrogen did not occur in our study regions.

**Figure 2.**
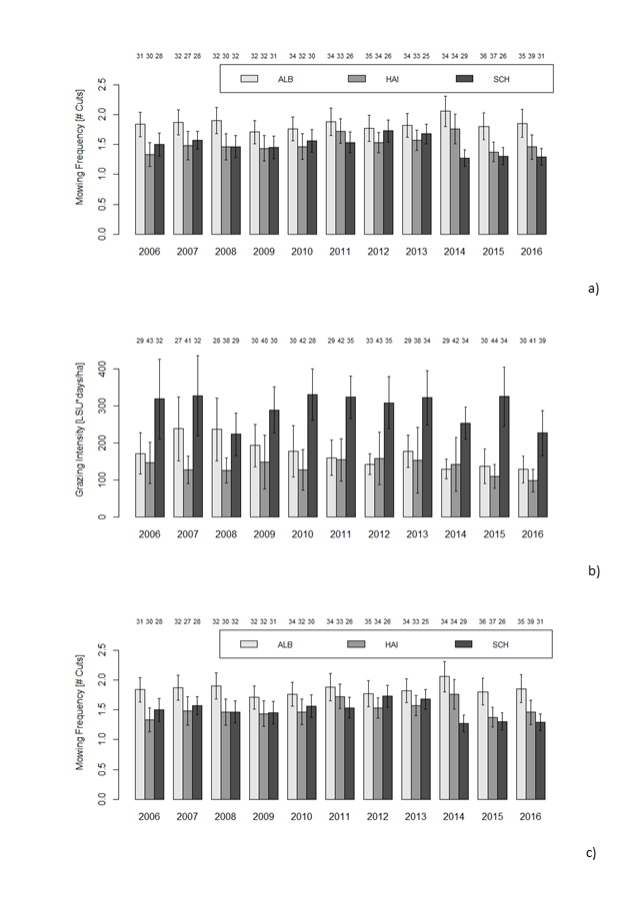
Annual means and standard error for a) mowing frequency in mown plots, i.e. the number of cuts per year, b) grazing intensity in grazed plots, in livestock unit days per hectare and year, calculated by multiplying the number of livestock by a conversion factor (see Table 3) and the number of grazing days and dividing the product by the size of the management unit and c) nitrogen fertilisation in fertilised plots, calculated as total nitrogen input in kg per hectare and year, in the three study regions of the Biodiversity Exploratories (light grey: Schwäbische Alb (ALB), grey: Hainich-Dün (HAI) and dark grey: Schorfheide-Chorin (SCH)). Only the subset of plots (out of 50 in each region), where the respective management was applied, are included in the figure panels (numbers above the bars).

Mean mowing frequency (number of cuts per year) across all 50 plots was between 0.6 and 1.5 and highest in the Alb, lower in Hainich and lowest in Schorfheide. Mowing frequency slightly increased in the Alb and Hainich, but decreased over the years in the Schorfheide. Within plots that were mown, mowing intensity was between 1.3 and 2 cuts per year and was highest in Alb, significantly higher than in Schorfheide (Fig. 2a). Mowing frequency within mown plots decreased over time in Schorfheide (Fig. 2a).

Grasslands were grazed by different types of livestock, most commonly cattle and sheep, but also horses and goats. Based on this information, the mean grazing intensity was then calculated by multiplying the livestock units ([LSU], (Table 2) with the duration of grazing per hectare [LSU days/ha]. Grazing intensity across all 50 plots in a region was on average between 120 and 200 livestock unit days per hectare in the Schorfheide, significantly higher than in the Alb (z = 3.177, p < 0.01) where yearly means were mostly below 100. Mean grazing intensity in Hainich was intermediate with yearly means below 150 (data not shown). In the Schorfheide, grazing intensity across the 50 plots increased slightly over time (z = 6.091, p < 0.0001, data not shown). In grazed plots, the annual grazing intensity per hectare ranged from 5 to 1644 livestock units x days. Mean grazing intensity in grazed plots was higher in the Schorfheide than in the other two regions, but due to high variability, differences between regions were not significant (p > 0.05, Fig. 2b). Within the grazed plots, grazing intensity in Schorfheide decreased over time (z = -3.270, p < 0.01, Fig. 2b), although the number of plots that were grazed were higher in the second half of the time series (Fig. 2b).

Fertilisation intensity across the 50 plots was highest in Hainich, with means mainly higher than 20 kg N*ha^-1^*yr^-1^, significantly higher than in the Schorfheide (z = 2.343, p < 0.05), where there was a significant decrease in fertilisation with time (z = -5.017, p < 0.001) and where yearly means dropped from 20 kg N ha^-1^ yr^-1^ to close to zero after 2013, which is largely due to a decrease in the number of fertilised plots to just two (Fig. 2c). Fertilisation in the Alb was intermediate (data not shown). Within fertilised plots, fertilisation ranged between 15 and 433 kg N ha^-1^ yr^-1^ and there were no differences between regions or changes over time (p > 0.05 in each case, Fig. 2c).

To summarise, there were significant differences between the regions in main grassland use, meadows in the Alb and pasture in Schorfheide and also in mean land use intensity of meadows, pastures or mown pastures. Changes over time were largely due to changes in the number of plots that were grazed, mown or fertilised, rather than to changes in mowing, grazing and fertilisation intensity within plots. In the Schorfheide, there was an overall decrease in land use intensity, due to increasing regulations in the biosphere reserve Schorfheide Chorin. In the Hainich, the number of fertilised plots decreased from 25 plots in 2006 to 12 plots in 2012 and then increased again to 22 in 2016 (Fig. 2c).

The management of grassland is decisively influenced by subsidies, such as agri-environmental measures (AEM) (Table 4). These AEMs are different within the federal states of Germany, having names such as MEKA or FAKT in Baden-Wuerttemberg and KULAP in Thuringia and Brandenburg, including single measures of different management aspects. The agri-environmental subsidy programmes aim to support environmental friendly and extensive production practices to protect natural resources and to preserve cultural landscapes. These can also be counted as disadvantage compensations and are co-financed by the EU, Germany and the respective federal state. Measures of these progammes determine guidelines regarding organic farming, the timing and type of mowing and grazing or restrictions, according to plant protection agents or fertiliser use (Table 4). Therefore, farmers do not make completely independent decisions by managing their grasslands but follow the regulations of the agri-environmental measures to receive subsidies for their land.Table 5 lists the agri-environmental measures applied for the single study plots for each year. The description of the coding of the agri-environmental measures is found as a legend in Table 6.

**Figure 3.**
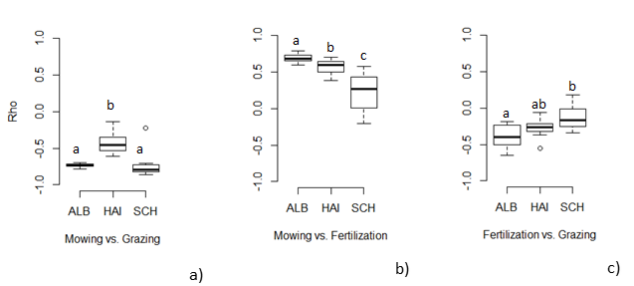
Boxplots showing the Spearman correlation coefficients (Rho) between the different grassland management components, calculated separately for each 11 years. a) mowing vs. grazing, b) mowing vs. fertilisation and c) fertilisation vs. grazing of the three different regions (ALB- Swabian Alb, HAI- Hainich, SCH- Schorfheide). Boxes with the same letters are not significantly different at p > 0.05 using pairwise Wilcoxon tests with Bonferroni correction.

In accordance with Blüthgen et al. 2012, mowing and fertilisation were correlated such that grasslands that were mown more frequently also received higher amounts of fertiliser. High grazing intensity was correlated with low mowing frequency such that intensively grazed grasslands were not mown and vice versa (Fig. 3a). Nevertheless, many grasslands were mown pastures, i.e. they are both grazed by varying types of livestock and mown using different practices. Timing of mowing and grazing was also variable between years. Correlations between mowing and grazing and between mowing and fertilisation, were stronger than those between fertilisation and grazing (Fig. 3). Correlations also differed between regions (Fig. 3). For example, in Hainich, there were weaker negative correlations between mowing and grazing compared to the Schwäbische Alb and Schorfheide.

The value of this dataset lies in the comprehensive and consistent description and characterisation of grassland management of 150 grassland plots over 11 years. Detailed accounting of land-use practices can only be achieved through intensive collaboration between land managers, land users and researchers, as done in our study. The accuracy of the answers given by farmers strongly determined the data quality. While in our study regions, all farmers had to keep records of management, mainly due to regulations of EU agricultural subsidies and cross-compliance obligations, the quality of the records still differed in some detail. To increase the accuracy of the data, members of the BE project additionally recorded data on grassland management over the years, such as cutting and fertilisation dates and maintenance activities. These observations were integrated when questionnaires were filled out together with the farmers. Values for organic fertilisation with slurry or liquid manure were probably less accurate than those for grazing and mowing, due to the fact that often no exact records existed for the amounts of material put on a particular site. Another source of uncertainty was the variation in N content of the material, which depended on many factors, for example, the livestock and the amount of added water etc. Here, we gave raw amounts of slurry and liquid manure, as well as the conversion factors to N per ha (Table 3).

The specific management data, presented here, have formed the basis for analyses of land use effects on the biodiversity and ecosystem functioning in grasslands (e.g. Allan et al. 2014, Manning et al. 2015, Klaus et al. 2018)Suppl. material 2. The data can be coupled with climate data and soil information to disentangle effects of management from effects of abiotic conditions (Smit et al. 2008). Our data show that ecologists, interested in the effects of land use on biodiversity and ecosystem functions, should pay closer attention to measurements of land use itself, because management can strongly vary between years with significant long-term effects on the target variables measured in any particular year (Klaus et al. 2011, Kleinebecker et al. 2018).

### Quality control

Quality assurance took place by checking the plausibility of the values by the grassland experts of the local teams in the following ways i) the answers of the land users were compared with own observations before entering the values in the data table. When uploading the data, the values were again checked ii) by the responsible person for the whole dataset looking at the single values and communicating uncertainties back to the interviewers. Further, unusual values were detected by auxiliary calculations, including minimum and maximum of these values and then double checked with the original hard copy version of the interview.

### Step description

The original interviews are stored in hard copies and the information is entered in the joint data table.

The data are stored on the Biodiversity-Exploratories Information System (BExIS) (http://doi.org/10.17616/R32P9Q) at https://www.bexis.uni-jena.de. This version is in German due to the annual survey of the land users being carried out in German. The interviews of each grassland site is entered in a default excel data sheet and transformed via a visual basic script before it is uploaded to the joint dataset in BExIS.

Daily backups at the BE repository ensures the storage of the actual version of the land use data table. After project end, all datasets are intended to be stored via GFBio in a domain-specific long term archive.

## Geographic coverage

### Description

The monitored 150 grassland plots are situated within three regions in Germany, with 50 plots in each region, covering a geographic gradient from the North-East (Schorfheide Chorin), Central Germany (Hainich Dün) and South-West (Schwäbische Alb). The grassland plots experience different land use according to the management.

## Taxonomic coverage

### Description

We did not collect any organisms. During the interviews, the land users provided us with information according to their grassland management.

## Temporal coverage

### Notes

We obtained land use data of grasslands between 2006 and 2016.

## Usage rights

### Use license

Other

### IP rights notes

The English version of the dataset is added as supplementary material.

The original, slightly extended, dataset is stored on the Biodiversity-Exploratories Information System (BExIS) (http://doi.org/10.17616/R32P9Q) at https://www.bexis.uni-jena.de. This version is in German due to the annual survey of the land users being carried out in German. Contact is possible via the Biodiversity Coordination Office (beo@senckenberg.de). Due to sensitive information, such as personal data, the original dataset is not publicly available. Data access can be given by individual request for access. Guidelines can be checked in the data agreement of the BE: https://www.bexis.uni-jena.de/PublicData/Files/PublicData-DataAgreement.txt.

## Data resources

### Data package title

BE_landuse_grassland_2006-2016.csv

### Number of data sets

1

### Data set 1.

#### Data set name

BE_landuse_grassland_2006-2016.csv

#### Number of columns

56

#### Description

The data table (628 KB) contains 1651 rows with records of eleven years on 150 grassland sites, including the variable headers and **116** (Suppl. material 1).

**Data set 1.** 

Column label	Column description
Study region	ALB = Schwäbische Alb, HAI = Hainich, SCH = Schorfheide
Year	Year of management
Date	Date of interview
PlotID	Experimental Plot IDs formatted as (A|H|S)EG with consecutive numbering. Abbreviations are: A = Schwäbische Alb, H = Hainich, S = Schorfheide, E = Experimental Plot, G = Grassland, e.g. AEG01
Drainage	Measure of drainage and the description of the method (free text)
StartDrainage	Starting year of grassland drainage if applicable
WaterLogging	Activities on water logging, e.g. for water regulation of fen soils
Agriculture1981	Use of grassland between 1981 to 2006, i.e. (temporal) conversion of grassland into arable land
SizeManagementUnit_ha	Size of the management unit in the survey year, often larger than the 50 x 50 m study plot itself
StartGrazing	Starting month of the first grazing period in the survey year
EndGrazing	End of the last grazing period in the survey year
Livestock1 (2-4)	Type of animal in first (second - fourth) grazing period
StartGrazingPeriod1 (2-4)	Starting month of first grazing period for livestock 1 (2-4)
EndGrazingPeriod1 (2-4)	Ending month of first grazing period for livestock 1 (2-4)
Cattle6months1 (2-4)	For Grazing period 1 (2-4): Number of cattle with an age up to 6 months (1 cattle up to 6 month = 0.3 LS* per day)
Cattle6-24months1 (2-4)	For Grazing period 1 (2-4): Number of cattle with an age between 6 months and 2 years (=0.6 LS*)
CattlePlus2years1 (2-4)	For Grazing period 1 (2-4): Number of cattle older than 2 years (= 1 LS*)
SheepGoat1year1 (2-4)	For Grazing period 1 (2-4): Number of sheep or goats with an age up to 1 year (= 0.05 LS*)
Pony1 (2-4)	For Grazing period 1 (2-4): Number of ponies and small horses (= 0.7 LS*)
Horse3years1 (2-4)	For Grazing period 1 (2-4): Number of horses up to 3 years (= 0.7 LS*)
HorsePlus3years1 (2-4)	For Grazing period 1 (2-4): Number of horses older than 3 years (= 1.1 LS*)
NbLivestock1 (2-4)	For Grazing period 1 (2-4): Total number of livestock
LivestockUnits1 (2-4)	For Grazing period 1 (2-4): Total sum of the livestock units
DayGrazing1 (2-4)	For Grazing period 1 (2-4): duration of grazing (in days)
GrazingArea1 (2-4)	For Grazing period 1 (2-4): size of area where livestock grazed
Mowing	Number of cuts per year
DateMowing1 (2-4)	Date of the first (second-fourth) cut
MowingMachine	Type of machine which was used for mowing e.g. rotarymower, doubleknife, mulcher
CutWidth_m	Cutting width of the mowing machine
CutHeight_cm	Cutting height above soil level of the mowing machine
DriveSpeed_kmh	Speed of the mowing machine, normally mean speed value is given
MowingConditioner	Presence of conditioner, i.e. did the mowing machine have a conditioner to improve drying of the clippings
Fertilisation	Addition of fertiliser (not including dung depositions by livestock during grazing a parcel)
NbFertilisation	Number of fertiliser applications per year
DateFertilisation1 (2-7)	Date of first (2nd-7th) fertiliser application
Manure_tha	Total amount of applied solid manure
Slurry_m3ha	Total amount of applied pig or cow slurry and biogas residues, respectively
DescFert	Description of applied organic fertiliser
orgNitrogen_kgNha	Amount of organic nitrogen applied
minNitrogen_kgNha	Amount of nitrogen applied, of mineral origin or the organic fertiliser mash from a bioethanol factory (see in DescFert)
totalNitrogen_kgNha	Sum of applied mineral and organic nitrogen [kg N/ha]
minPhosphorus_kgPha	Amount of phosphorus applied [kg P_2_O_5_/ha], of mineral origin or mash from a bioethanol factory (not given for other organic fertilisers)
minPotassium_kgKha	Amount of potassium applied [kg K_2_O/ha], of mineral origin or mash from a bioethanol factory (not given for other organic fertilisers)
Sulphur_kgSha	Total amount of applied Sulphur [kg S/ha]
Maintenance	Presence of maintenance measures
Levelling	Maintenance to break up matted grass covers
DateLevelling	Maintenance: date of levelling
Rolling	Maintenance: rolling to level unevenness
DateRolling	Maintenance: date of rolling
Mulching	Partial mulching on some spots, e.g. rank patches. The material remains on site after mowing. We consider this not as a mowing event, as only a small part of the area is treated.
DateMulching	Date of partial mulching
ShrubClearance	Clearance to avoid shrub encroachment. We consider this not as a mowing event, as only individual shrubs are targeted.
DateScrubCl	Date of shrub clearance
PlantProtectionAgent	Pesticide use: pesticides and herbicides. As pesticides in grasslands are very rare and only used for spot treatment, we do not have further information on this treatment.
Seeds	Seed addition
DescSeeds	Description of usage of the sowing

## Supplementary Material

7C2A1598-D96E-59DA-97D6-60F4D50A2F0B 10.3897/BDJ.7.e36387.suppl1 Supplementary material 1 BE_landuse_grassland_2006-2016.csv Data type: utf 8 - txt
Brief description: The present dataset summarises management information collected from 2006 to 2016 for 150 grassland plots in three different regions of Germany. Data are based on annual interviews of the respective farmers, land owners or tenants involved in land management activity, using a standardised questionnaire.
Standardisation of missing values:
“NA” - if not known,
“0” if something was counted but was zero (e.g. no mowing or no cows or no maintenance). Some dates of maintenance or fertilisation are set to "0". For example, in case maintenance measurements were applied on that plot during the year but not the specific one, i.e. mulching was applied and is listed with specific mulching date, but no levelling took place, therefore the levelling date is set to "0" instead of "-1" when generally no maintenance measures were carried out on that plot within the year.
“-1” if not possible, for example, if no mowing a “-1” has been given for the question about mowing machine.
File: oo_338774.txt https://binary.pensoft.net/file/338774 Juliane Vogt, Valentin H. Klaus, Ralf Lauterbach, Niclas Otto, Uta Schumacher, Cornelia Fürstenau, Katrin Lorenzen, Andreas Ostrowski, Wolfgang W. Weisser

0349DD1C-1883-54B0-8BA8-8E996FC221ED 10.3897/BDJ.7.e36387.suppl2 Supplementary material 2 Bibliography of the land use index Data type: Text
Brief description: This library shows the citations of the LUI developed by Brüthgen et al. 2012.
File: oo_330477.txt https://binary.pensoft.net/file/330477 Juliane Vogt

## Figures and Tables

**Table 1.** Overview of all variables of the data set: BE_landuse_grassland_2006-2016.csv received from the management questionnaire.

**Variable**	**Type of data**	**Units**	**Range of numeric variables** **(min-max)**	**Description (English)**
ID	Text	-	-	Unique identifier composed of the columns PlotID and Year
Study region	Text		-	ALB = Schwäbische Alb HAI = Hainich SCH = Schorfheide
Year	Integer	yyyy	-	Year of management
Date	Date	dd.mm.yyyy	-	Date of interview
PlotID	Text	-	-	Experimental Plot IDs formatted as (A|H|S)EG with consecutive numbering. Abbreviations are: A = Schwäbische Alb, H = Hainich, S = Schorfheide E = Experimental Plot G = Grassland, e.g. AEG01
Drainage	Text	-	-	Measure of drainage and the description of the method (free text)
StartDrainage	Integer	yyyy	-	Starting year of grassland drainage, if applicable
WaterLogging	Boolean	yes/no	-	Activities on water logging, e.g. for water regulation of fen soils
Agriculture1981	Boolean	yes/no	-	Use of grassland between 1981 to 2006, i.e. (temporal) conversion of grassland into arable land
SizeManagementUnit_ha	Numeric	ha	0.49-187.1	Size of the management unit in the survey year, often larger than the 50 x 50 m study plot itself
***Grazing***				
StartGrazing	Text	Month	-	Starting month of the first grazing period in the survey year
EndGrazing	Text	Month	-	End of the last grazing period in the survey year
Livestock1	Text		-	Type of animal in first grazing period
StartGrazingPeriod1	Text	month	-	Starting month of first grazing period for livestock 1
EndGrazingPeriod1	Text	month	-	Ending month of first grazing period for livestock 1
Livestock, Start/End GrazingPeriod 2-4 …			-	Identical information for grazing periods 2-4, if applicable.
Cattle6months1	Integer		0-95	For Grazing period 1: Number of cattle with an age up to 6 months (cattle up to 6 months = 0.3 LS* per day)
Cattle6-24months1	Integer		0-200	For Grazing period 1: Number of cattle with an age between 6 months and 2 years (= 0.6 LS*)
CattlePlus2years1	Integer		0-300	For Grazing period 1: Number of cattle older than 2 years (= 1 LS*)
SheepGoat1year1	Integer		0-1000	For Grazing period 1: Number of sheep or goats with an age up to 1 year (= 0.05 LS*)
SheepGoatPlus1year1	Integer		0-1500	For Grazing period 1: Number of sheep or goats older than 1 year (= 0.1 LS*)
Pony1	Integer		0-400	For Grazing period 1: Number of ponies and small horses (= 0.7 LS*)
Horse3years1	Integer		0-4	For Grazing period 1: Number of horses up to 3 years (= 0.7 LS*)
HorsePlus3years1	Integer		0-46	For Grazing period 1: Number of horses older than 3 years (= 1.1 LS*)
NbLivestock1	Integer		0-2500	For Grazing period 1: Total number of livestock
LivestockUnits1	Numeric	Number of livestock x conversion factor	0-1814	For Grazing period 1: Total sum of the livestock units
DayGrazing1	Integer	days	0-365	For Grazing period 1: duration of grazing (in days)
GrazingArea1	Numeric	ha	0-148.5	For Grazing period 1: size of area where livestock grazed
***Numeric variables for grazing 2-4***…For Grazing periods 2-4 see description of grazing period 1				
Cattle6months2			0-73	
Cattle6-24months2			0-103	
CattlePlus2years2			0-120	
SheepGoat1year2			0-600	
SheepGoatPlus1year2			0-1200	
Pony2			0	
Horse3years2			0	
HorsePlus3years2			0-18	
NbLivestock2			0-1200	
LivestockUnits2			0-144	
DayGrazing2			0-165	
GrazingArea2			0-148.5	
Cattle6months3			0-72	
Cattle6-24months3			0-103	
CattlePlus2years3			0-120	
SheepGoat1year3			0-820	
SheepGoatPlus1year3			0-1300	
Pony3			0	
Horse3years3			0	
HorsePlus3years3			0-25	
NbLivestock3			0-1340	
LivestockUnits3			0-145.5	
DayGrazing3			0-127	
GrazingArea3			0-196.69	
Cattle6months4			0-72	
Cattle6-24months4			0-81	
CattlePlus2years4			0-84	
SheepGoat1year4			0-600	
SheepGoatPlus1year4			0-900	
Pony4			0	
Horse3years4			0	
HorsePlus3years4			0-16	
NbLivestock4			0-900	
LivestockUnits4			0-103.6	
DayGrazing4			0-76	
GrazingArea4			0-148.5	
TotalGrazing_LSUdha	Numeric	#Livestock*days /ha	0-1644.17	Total sum of the grazing intensity for all grazing periods
SupplementaryFeeding	Boolean	yes/no	-	Additional fodder supply for the livestock
DescFeeding	Text		-	Type and amount of supplementary fodder
***Mowing***				
Mowing	Integer	1/year	0-4	Number of cuts per year
DateMowing1	Date	dd.mm.yyyy	-	Date of the first cut
DateMowing2-4…	Date	dd.mm.yyyy	-	Dates of the second to fourth cut, if applicable
MowingMachine	Text		-	Type of machine which was used for mowing, e.g. rotarymower, doubleknife, mulcher
CutWidth_m	Numeric	m	0-12	Cutting width of the mowing machine
CutHeight_cm	Integer	cm	0-15	Cutting height above soil level of the mowing machine
DriveSpeed_kmh	Integer	km/h, (mean)	0-20	Speed of the mowing machine, normally mean speed value is given
MowingConditioner	Boolean	yes/no	-	Presence of conditioner, i.e. did the mowing machine have a conditioner to improve drying of the clippings
***Fertilisation***				
Fertilisation	Boolean	yes/no	-	Addition of fertiliser (not including dung depositions by livestock during grazing a parcel)
NbFertilisation	Integer		0-7	Number of fertiliser applications per year
DateFertilisation1	Date	dd.mm.yyyy	-	Date of first fertiliser application
DateFertilisation2-7…	Date	dd.mm.yyyy	-	Date of 2nd to 7th fertiliser applications
Manure_tha	Numeric	t/ha	0-40	Total amount of applied solid manure
Slurry_m3ha	Numeric	m³/ha	0-80	Total amount of applied pig or cow slurry and biogas residues, respectively.
DescFert	Text		-	Description of applied organic fertiliser
orgNitrogen_kgNha	Numeric	kg/ha	0-371	Amount of organic nitrogen applied
minNitrogen_kgNha	Numeric	kg/ha	0-170	Amount of nitrogen applied, of mineral origin or the organic fertiliser mash from a bioethanol factory (see in DescFert)
totalNitrogen_kgNha	Numeric	Kg /ha	0-433	Sum of applied mineral and organic nitrogen [kg N/ha]
minPhosphorus_kgPha	Numeric	kg/ha	0-350	Amount of phosphorus applied [kg P_2_O_5_/ha], of mineral origin or mash from a bioethanol factory (not given for other organic fertilisers)
minPotassium_kgKha	Numeric	kg/ha	0-100	Amount of potassium applied [kg K_2_O/ha], of mineral origin or mash from a bioethanol factory (not given for other organic fertilisers)
Sulphur_kgSha	Numeric	kg/ha	0-25	Total amount of applied Sulphur [kg S/ha]
***Maintenance***				
Maintenance	Boolean	yes/no	-	Presence of maintenance measures
Levelling	Text		0-4	Maintenance to break up matted grass covers
DateLevelling	Date	dd.mm.yyyy	-	Maintenance: date of levelling
Rolling	Text		0-2	Maintenance: rolling to level unevenness
DateRolling	Date	dd.mm.yyyy	-	Maintenance: date of rolling
Mulching	Text		0-4	Partial mulching on some spots, e.g. rank patches. The material remains on site after mowing. We consider this not as a mowing event as only a small part of the area is treated.
DateMulching	Date	dd.mm.yyyy	-	Date of partial mulching
ShrubClearance	Text	-	0-1	Clearance to avoid shrub encroachment. We consider this not as a mowing event as only individual shrubs are targeted.
DateScrubCl	Date	dd.mm.yyyy	-	Date of shrub clearance
PlantProtectionAgent	Boolean	yes/no	-	Pesticide use: pesticides and herbicides. As pesticides in grasslands are very rare and only used for spot treatment, we do not have further information on this treatment.
Seeds	Boolean	yes/no	-	Seed addition
DescSeeds	Text	-	-	Description of usage of the sowing

^*^LS – Livestock

**Table 2.** Livestock units derived from the type and age of livestock (Chamber of Agriculture Nordrhein-Westfalen 2018).

**Grazing species**	**Age**	**Livestock units (LSU)**
Cattle	< 6 months	0.3
Cattle	6 months-2 years	0.6
Cattle	> 2 years	1
Sheep and goats	< 1 year	0.05
Sheep and goats	> 1 year	0.1
Ponies and small horses	-	0.7
Horses	< 3 years	0.7
Horses	> 3 years	1.1

**Table 3.** Nitrogen input conversion factor of manure and slurry.

**Type of manure (t/ha)**	**Conversion Factor for total Nitrogen [kg/t**]	**Literature and Notes**
Cattle	5.6	LWK (Chamber of Agriculture) Nordrhein-Westfalen (2014), own measurements analysed by LUFA Nord-West (Agricultural Investigation and Research Institute - accredited laboratory of the Chamber of Agriculture in Niedersachen) (2017)
Horse	4.9	
Sheep	8.13	
**Type of slurry (m³/ha)**	**Total Nitrogen [kg/m**³]	
Cattle	3.85 (3.2-4.5)	Mean values of slurry ranges were used. (LWK Nordrhein-Westfalen(2014))
Pig	5.4 (4.3-6.5)	
Mixed	4.45 (4.0-4.9)	
Biogas / Digestate	4.4	LWK Baden-Württemberg (2012)

**Table 4.** The requirements of single agri-environmental measures (MEKA/FAKT for Baden-Wuerttemberg and KULAP for Thuringia and Brandenburg) are characterised by the subprogramme designation listed for every region (ALB- Swabian Alb, HAI- Hainich, SCH- Schorfheide). The abbreviations (R)LSU mean (roughage consuming) livestock units having a livestock-dependent conversion from LSU to RLSU: 1 LSU equals the RLSU for sheep or goat (0.7), horse (0.5), cattle (1).

**Agri-environmental measures**	**ALB (MEKA, FAKT)**	**HAI (KULAP)**	**SCH (KULAP)**
**Requirements**			
Difficult management due to slope of ≥ 25%	N-B3		
Adapted, extensive management of biotope (§32 nature conservation)	N-G1.1, B4		
FFH: lowlands- and mountain-meadows	B5		
Conservation of meadow orchards (eligible up to max 100 trees/ha)	C1		
Low-nutrient and dry habitats (biotopes maintenance by grazing)		N21, G21	
Low-nutrient and dry habitats (biotopes maintenance by mowing)		N31	
Wet meadows		N23	
Eligible landscape (e.g. Natura 2000)			413A, 423B, 613A, 663
Sheep farming and difficult terrain		N25	
Difficult conditions (regarding terrain, specific management)		G31, G33, G53	
Location of valuable genetic plants		L4	
Compensatory allowance of disadvantaged sites			33
**Organic farming**			
Farm is managed according to EU eco-regulation	N-D2, D2		
Introduction or retention of ecological management of the farm		L1, Ö2	773, 673,882
Retention of ecological management - compensatory allowance			623 A,B,C,D
**In general**			
Main fodder site	B1.2		
Min 5% of eligible site managed after 15 June	N-B1, N-B3		
Promoting of endangered livestock breeds	C3		
No reduction of permanent grassland of the farm		N25	
Management plan according to nature conservation authority		N231, N31, G21, G31, G33, G53	663
Fodder sites are managed at least once per year by grazing or mowing			413A, 423B, 613A, 663, 673
Management after 1 July			812C
**Grazing**			
Livestock min 0.3 LSU/ha on agriculture area		Ö2	
Livestock max 2 LSU/ha on agriculture area	N-B1, N-B3		773, 673
Livestock min 0.5 RLSU/ha fodder area		L4	
Livestock min 0.3 RLSU/ha fodder area	N-B2, B1.1, B1.2		311A, 311C, 773, 673, 661, 411
Livestock max 1.4 RLSU/ ha			311A, 311C, 773, 673, 661, 411
Livestock max 1.4 LSU/ ha	N-B2, B1.1		311A, 311C
At least one grazing per year		N25	
At least one grazing per year. First grazing period by cattle/horses or sheep/goats		G21	
At least one grazing per year. First grazing period by sheep/goats		G33, G53	
At least one management per year (grazing or mowing and harvesting of the yield) before 15 October			411, 661
Maintenance measures after grazing	N-B1, N-B2, N-B3		
Grazing by cattle/horses with 0.3-1 LSU/ha		N211	
Grazing by cattle/horses with permanent grazing or at least from 2 May to 15 October		G31	
Grazing by sheep or goats with a min 0.5 LSU/ha		N213, N25, G33, G53	
Grazing 0.3-1 LSU/ha		N231	
Max 1.5 LSU/ha*d until 1 July		N231	
First management of the year: at least 80% of area by grazing (up to 20% by mowing)		N231	
First management mowing: grazing possible at least 7 weeks after the first cut		N31	
No supplementary feeding		N233	
No supplementary feeding between 1 May and 15 October		G21, G31, G33, G53	
**Mowing**			
First management mowing and harvesting the yield		N31	
Up to 2 cuts with a time lag of at least 7 weeks		N31	
First mowing not before 15 August on min 5% of the area		N31	
No mowing before 16 June			313A
No mowing before 1 July			313B
Post grazing mowing not before 1 July		N231, G31, G33, G53	
Cut height 10 cm			313B, 763
**Indicator plant species**			
Abundance of at least 4 indicator plant species out of 28 specific forbs	N-B4	L4	
Abundance of at least 6 indicator plant species out of 30 specific plants	B3.2	G11	
Abundance of at least 7 indicator plant species out of specific plants	B5		
**Fertilisation**			
No mineral nitrogen fertilisation	B1.1		
No mineral or organic nitrogen fertilisation	B1.2		
No slurry fertilisation			311C
No fertilisation			811A
No chemical-synthetic fertiliser or plant protection agent within the farm	D1		311A, 311C, 661, 411
No chemical-synthetic fertiliser or plant protection agent on eligible areas		N231, N25; N31	
No fertiliser or plant protection agent		G21, G31, G33	
**Documentation**			
Slurry records (amount, date) for eligible areas	N-B1, N-B3		
Fertilisation and management records for eligible areas	N-B4		
Fertilisation and mowing records for eligible areas	B3.2		
Fertilisation and plant protection agent records for all grasslands of the farm	B1.2		
Records via Thuringian grassland card for eligible areas		N231, N25, N31, G21, G31, G33, G53	
**Restrictions /measures not taken**			
No ploughing, only seed addition	B1.1, B1.2, B3.2		
No ploughing on eligible areas			
No ploughing on farm	N-B1, N-B2, N-B3, N-B4, N-D2		413A, 423B, 411, 661
No irrigation or melioration	N-B2, B1.1, B1.2	G21, G31, G33, G53	
No extensive usage of plant protection agents	N-B1, N-B2, N-B3, N-B4, B1.1, B1.2		
No maintenance measures, mowing or seed addition between 1 April and 30 June		G21, G31, G33, G53	
No upturning or limbering tillage		G21, G31, G33, G53	

**Table 5.** Study plots with the geographical coordinates and the coding of the agri-environmental measures. The description of coding is found in the legend of Table 6.

EP_Plot_ID	Explo	Latitude	Longitude	2006	2007	2008	2009	2010	2011	2012	2013	2014	2015	2016
AEG1	ALB	48.4	9.34	k 0 n 0	k 0 n 0	k 0 n 0	k 0 n 0	k 0 n 0	k 0 n 0	k 0 n 0	k 0 n 0	k 0 n 0	k 0 n 0	k 0 n 0
AEG10	ALB	48.38	9.21	o 0 n A5	o 0 n A5	o 0 n A5	o 0 n A5	o 0 n A5	o 0 n A5	o 0 n A5	o 0 n A5	o 0 n A5	k 0 n 0	k 0 n 0
AEG11	ALB	48.49	9.35	k 0 n 0	k 0 n 0	k 0 n 0	k 0 n 0	k 0 n 0	k 0 n 0	k 0 n 0	k 0 n 0	k 0 n 0	k 0 n 0	k 0 n 0
AEG12	ALB	48.39	9.35	k 0 n 0	k 0 n 0	k 0 n 0	k 0 n 0	k 0 n 0	k 0 n 0	k 0 n 0	k 0 n 0	k 0 n 0	k 0 n 0	k 0 n 0
AEG13	ALB	48.39	9.36	k 0 n 0	k 0 n 0	k 0 n 0	k 0 n 0	k 0 n 0	k 0 n 0	k 0 n 0	k 0 n 0	k 0 n 0	k 0 n 0	k 0 n 0
AEG14	ALB	48.38	9.52	k 0 n 0	k 0 n 0	k 0 n 0	k 0 n 0	k 0 n 0	k 0 n 0	k 0 n 0	k 0 n 0	k 0 n 0	k 0 n 0	k 0 n 0
AEG15	ALB	48.49	9.45	k 0 n 0	k 0 n 0	k 0 n 0	k 0 n 0	k 0 n 0	k 0 n 0	k 0 n 0	k 0 n 0	k 0 n 0	k 0 n 0	k 0 y A12
AEG16	ALB	48.4	9.46	o c y A4/A5	o c y A4/A5	o c y A4/A5	o c y A4/A5	o c y A4/A5	o c y A4/A5	o c y A4/A5	o c y A4/A5	o c y A4/A5	o c y A14/11/A12	o c y A14/A16/A12
AEG17	ALB	48.4	9.52	o a y 0	o a y 0	o a y 0	o a y 0	o a y 0	o a y 0	o a y 0	o a y 0	o a y 0	o c 0 0	o c 0 0
AEG18	ALB	48.38	9.52	k 0 n 0	k 0 n 0	k 0 n 0	k 0 n 0	k 0 n 0	k 0 n 0	k 0 n 0	k 0 n 0	k 0 n 0	k 0 n 0	k 0 n 0
AEG19	ALB	48.4	9.45	k 0 n 0	k 0 n 0	k 0 n 0	k 0 n 0	k 0 n 0	k 0 n 0	k 0 n 0	k 0 n 0	k 0 n 0	k 0 n 0	k 0 n 0
AEG2	ALB	48.38	9.47	k 0 n 0	k 0 n 0	k 0 n 0	k 0 n 0	k 0 n 0	k 0 n 0	k 0 n 0	k 0 n 0	k 0 n 0	k 0 n 0	k 0 n 0
AEG20	ALB	48.49	9.36	k 0 n 0	k 0 n 0	k 0 n 0	k 0 n 0	k 0 n 0	k 0 n 0	k 0 n 0	k 0 n 0	k 0 n 0	k 0 n 0	k 0 n 0
AEG21	ALB	48.44	9.36	o 0 y A5	o 0 y A5	o 0 y A5	o 0 y A5	o 0 y A5	o 0 y A5	o 0 y A5	o 0 y A5	0 0 0 0	k 0 n 0	k 0 n 0
AEG22	ALB	48.4	9.51	k 0 y A1	k 0 y A1	k 0 y A1	k 0 y A1	k 0 y A1	k 0 y A1	k 0 y A1	k 0 y A1	k 0 y A1	k 0 y A7/A10	k 0 y A7/A10
AEG23	ALB	48.42	9.51	k 0 y 0	k 0 y 0	k 0 y 0	k 0 y 0	k 0 y 0	k 0 y 0	k 0 y 0	k 0 y 0	k 0 y 0	o c y A12	o c y A12
AEG24	ALB	48.4	9.49	k 0 y 0	k 0 y 0	k 0 y 0	k 0 y 0	k 0 y 0	k 0 y 0	k 0 y 0	k 0 y 0	k 0 y 0	k 0 y A15	k 0 y A15
AEG25	ALB	48.4	9.26	k 0 y 0	k 0 y 0	k 0 y 0	k 0 y 0	k 0 y 0	k 0 y 0	k 0 y 0	k 0 y 0	k 0 y 0	k 0 y A19	k 0 y A19
AEG26	ALB	48.4	9.4	k 0 y 0	k 0 y 0	k 0 y 0	k 0 y 0	k 0 y 0	k 0 y 0	k 0 y 0	k 0 y 0	k 0 y 0	k 0 y A15/A19	k 0 y A15/A19
AEG27	ALB	48.42	9.48	k 0 y 0	k 0 y 0	k 0 y 0	k 0 y 0	k 0 y 0	k 0 y 0	k 0 y 0	k 0 y 0	k 0 y 0	k 0 y A15/A17/A20/A19	k 0 y A15/A17/A20/A19
AEG28	ALB	48.46	9.49	k 0 y 0	k 0 y 0	k 0 y 0	k 0 y 0	k 0 y 0	k 0 y 0	k 0 y 0	k 0 y 0	k 0 y 0	k 0 y A15	k 0 y A15
AEG29	ALB	48.42	9.36	k 0 y 0	k 0 y 0	k 0 y 0	k 0 y 0	k 0 y 0	k 0 y 0	k 0 y 0	k 0 y 0	k 0 y 0	k 0 y A9/A11	k 0 y A9/A11
AEG3	ALB	48.41	9.53	o a y A5	o a y 0	o a y 0	o a y 0	o a y 0	o a y 0	o a y 0	o a y 0	o c y 0	o c y A12	o c y A12
AEG30	ALB	48.46	9.46	k 0 y 0	k 0 y 0	k 0 y 0	k 0 y 0	k 0 y 0	k 0 y 0	k 0 y 0	k 0 y 0	k 0 y 0	k 0 y A15	k 0 y A15
AEG31	ALB	48.46	9.46	k 0 y 0	k 0 y 0	k 0 y 0	k 0 y 0	k 0 y 0	k 0 y 0	k 0 y 0	k 0 y 0	k 0 y 0	k 0 y A15	k 0 y A15
AEG32	ALB	48.47	9.49	k 0 y 0	k 0 y 0	k 0 y 0	k 0 y 0	k 0 y 0	k 0 y 0	k 0 y 0	k 0 y 0	k 0 y 0	k 0 y A15	k 0 y A15
AEG33	ALB	48.45	9.49	k 0 y 0	k 0 y 0	k 0 y 0	k 0 y 0	k 0 y 0	k 0 y 0	k 0 y 0	k 0 y 0	k 0 y 0	k 0 y A15/A11	k 0 y A15/A11
AEG34	ALB	48.46	9.5	k 0 y 0	k 0 y 0	k 0 y 0	k 0 y 0	k 0 y 0	k 0 y 0	k 0 y 0	k 0 y 0	k 0 y 0	k 0 y A15	k 0 y A15
AEG35	ALB	48.48	9.29	k 0 n 0	k 0 n 0	k 0 n 0	k 0 n 0	k 0 n 0	k 0 n 0	k 0 n 0	k 0 n 0	k 0 n 0	k 0 n 0	k 0 n 0
AEG36	ALB	48.48	9.3	k 0 n 0	k 0 n 0	k 0 n 0	k 0 n 0	k 0 n 0	k 0 n 0	k 0 n 0	k 0 n 0	k 0 n 0	k 0 n 0	k 0 n 0
AEG37	ALB	48.4	9.41	k 0 n 0	k 0 n 0	k 0 n 0	k 0 n 0	k 0 n 0	k 0 n 0	k 0 n 0	k 0 n 0	k 0 n 0	k 0 n 0	k 0 n 0
AEG38	ALB	48.44	9.43	k 0 y 0	k 0 y 0	k 0 y 0	k 0 y 0	k 0 y 0	k 0 y 0	k 0 y 0	k 0 y 0	k 0 y 0	k 0 n 0	k 0 n 0
AEG39	ALB	48.39	9.43	k 0 n 0	k 0 n 0	k 0 n 0	k 0 n 0	k 0 n 0	k 0 n 0	k 0 n 0	k 0 n 0	k 0 n 0	k 0 n 0	k 0 n 0
AEG4	ALB	48.38	9.42	k 0 n 0	k 0 n 0	k 0 n 0	k 0 n 0	k 0 n 0	k 0 n 0	k 0 n 0	k 0 n 0	k 0 n 0	k 0 n 0	k 0 n 0
AEG40	ALB	48.41	9.57	k 0 y A1/A19	k 0 y A1/A19	k 0 y A1/A19	k 0 y A1/A19	k 0 y A1/A19	k 0 y A1/A19	k 0 y A1/A19	k 0 y A1/A19	k 0 y A1/A19	k 0 n 0	k 0 n 0
AEG41	ALB	48.37	9.4	k 0 y A2	k 0 y A2	k 0 y A2	k 0 y A2	k 0 y A2	k 0 y A2	k 0 y A2	k 0 y A2	k 0 y A2	k 0 n 0	k 0 n 0
AEG42	ALB	48.4	9.38	k 0 y 0	k 0 y 0	k 0 y 0	k 0 y 0	k 0 y 0	k 0 y 0	k 0 y 0	k 0 y 0	k 0 y 0	k 0 n 0	k 0 n 0
AEG43	ALB	48.41	9.54	k 0 y 0	k 0 y 0	k 0 y 0	k 0 y 0	k 0 y 0	k 0 y 0	k 0 y 0	k 0 y 0	k 0 n 0	k 0 y A8	k 0 y A8
AEG44	ALB	48.38	9.43	k 0 y 0	k 0 y 0	k 0 y 0	k 0 y 0	k 0 y 0	k 0 y 0	k 0 y 0	k 0 y 0	k 0 y 0	k 0 n 0	k 0 n 0
AEG45	ALB	48.4	9.46	o 0 y A5	o 0 y A5	o 0 y A5	o 0 y A5	o 0 y A5	o 0 y A5	o 0 y A5	o 0 y A5	o 0 y A5	o 0 y A13	o 0 y A13
AEG46	ALB	48.4	9.43	o c y A5	o c y A5	o c y A5	o c y A5	o c y A5	o c y A5	o c y A5	o c y A5	o c y A5	o c y A16/A18	o c y A16/A18
AEG47	ALB	48.42	9.45	k 0 y A5/A1/A6	k 0 y A5/A1/A6	k 0 y A5/A1/A6	k 0 y A5/A1/A6	k 0 y A5/A1/A6	k 0 y A5/A1/A6	k 0 y A5/A1/A6	k 0 y A5/A1/A6	k 0 n 0	k 0 y A19	k 0 y A19
AEG48	ALB	48.42	9.5	k 0 y 0	k 0 y 0	k 0 y 0	k 0 y 0	k 0 y 0	k 0 y 0	k 0 y 0	k 0 y 0	k 0 y 0	k 0 y A15/A17/A20/A19	k 0 y A15/A17/A20/A19
AEG49	ALB	48.46	9.5	k 0 y 0	k 0 y 0	k 0 y 0	k 0 y 0	k 0 y 0	k 0 y 0	k 0 y 0	k 0 y 0	k 0 y 0	k 0 y A15	k 0 y A15
AEG5	ALB	48.4	9.44	k 0 n 0	k 0 n 0	k 0 n 0	k 0 n 0	k 0 n 0	k 0 n 0	k 0 n 0	k 0 n 0	k 0 n 0	k 0 n 0	k 0 n 0
AEG50	ALB	48.41	9.47	k 0 n 0	k 0 n 0	k 0 n 0	k 0 n 0	k 0 n 0	k 0 n 0	k 0 n 0	k 0 n 0	k 0 n 0	k 0 n 0	k 0 n 0
AEG6	ALB	48.4	9.44	k 0 n 0	k 0 n 0	k 0 n 0	k 0 n 0	k 0 n 0	k 0 n 0	k 0 n 0	k 0 n 0	k 0 n 0	k 0 n 0	k 0 n 0
AEG7	ALB	48.39	9.38	k 0 y 0	k 0 y 0	k 0 y 0	k 0 y 0	k 0 y 0	k 0 y 0	k 0 y 0	k 0 y 0	k 0 y 0	k 0 y A19/A15	k 0 y A19/A15
AEG8	ALB	48.42	9.49	k 0 y 0	k 0 y 0	k 0 y 0	k 0 y 0	k 0 y 0	k 0 y 0	k 0 y 0	k 0 y 0	k 0 y 0	k 0 y A15/A11	k 0 y A15/A11
AEG9	ALB	48.39	9.5	k 0 y 0	k 0 y 0	k 0 y 0	k 0 y 0	k 0 y 0	k 0 y 0	k 0 y 0	k 0 y 0	k 0 y 0	k 0 y A15/A17/A20/A19	k 0 y A15/A17/A20/A19
HEG1	HAI	50.97	10.41	k 0 n 0	k 0 n 0	k 0 n 0	k 0 n 0	k 0 n 0	k 0 n 0	k 0 n 0	k 0 n 0	k 0 n 0	k 0 n 0	k 0 n 0
HEG10	HAI	51.28	10.45	k 0 y 0	k 0 y 0	o 0 y 0	o i y H4	o i y H4	o i y H4	o a y H4	o a y H4	o a y H4	o a y H12	o a y H12
HEG11	HAI	51.28	10.46	k 0 y 0	k 0 y 0	o 0 y 0	o i y H4	o i y H4	o i y H4	o a y H4	o a y H4	o a y H4	o a y H12	o a y H12
HEG12	HAI	51.08	10.58	k 0 y 0	k 0 y 0	k 0 y 0	k 0 y H4	k 0 y H5	k 0 y H5	k 0 y H5	k 0 y H5	k 0 y H5	k 0 y H13	k 0 n 0
HEG13	HAI	51.26	10.38	k 0 y 0	k 0 y 0	k 0 y 0	k 0 y 0	k 0 y 0	k 0 y 0	k 0 y 0	k 0 y 0	k 0 y 0	k 0 n 0	k 0 y 0
HEG14	HAI	51.29	10.44	k 0 y 0	k 0 y 0	k 0 y 0	k 0 y H4	k 0 y 0	k 0 y H5	k 0 y H5	k 0 y H5	k 0 y H5	k 0 y H13	k 0 y H13
HEG15	HAI	51.07	10.49	k 0 y H2	k 0 y H2	k 0 y H2	k 0 n 0	k 0 n 0	k 0 n 0	k 0 y 0	k 0 y 0	k 0 y 0	k 0 y 0	k 0 y 0
HEG16	HAI	51.03	10.46	k 0 y H3	k 0 y H3	k 0 y H3	k 0 y H8	k 0 y H8	k 0 y H8	k 0 y H8	k 0 y H8	k 0 y H8	k 0 y H16	k 0 y H16
HEG17	HAI	51.07	10.47	k 0 y 0	k 0 y 0	k 0 y 0	k 0 y H8	k 0 y H8	k 0 y H8	k 0 y H8	k 0 y H8	k 0 y H8	k 0 y H16	k 0 y H17
HEG18	HAI	51.28	10.42	k 0 y 0	k 0 y 0	k 0 y 0	k 0 y 7	k 0 y 0	k 0 y H8	k 0 y H8	k 0 y H8	k 0 y H8	k 0 y H16	k 0 y H16
HEG19	HAI	51.07	10.47	k 0 y 0	k 0 y 0	k 0 y 0	k 0 y H8	k 0 y 0	k 0 y H8	k 0 y H8	k 0 y H8	k 0 y H8	k 0 y H16	k 0 y H17
HEG2	HAI	51	10.43	k 0 n 0	k 0 n 0	k 0 n 0	k 0 n 0	k 0 n 0	k 0 n 0	k 0 y 0	k 0 n 0	k 0 n 0	k 0 n 0	k 0 n 0
HEG20	HAI	51.22	10.37	k 0 y 0	k 0 y 0	k 0 y 0	k 0 y 0	k 0 y 0	k 0 y H8	k 0 y H8	k 0 y H9	k 0 y H8	k 0 y H16	k 0 y H16
HEG21	HAI	51.19	10.75	k 0 y 0	k 0 y 0	k 0 y 0	k 0 y H8	k 0 y H8	k 0 y H8	k 0 y H8	k 0 y H8	k 0 y H8	k 0 y H16	k 0 y H16
HEG22	HAI	51.03	10.32	k 0 y 0	k 0 y 0	k 0 y 0	k 0 y H9	k 0 y H9	k 0 y H9	k 0 y H9	k 0 y H9	k 0 y H9	k 0 y H13	k 0 y 0
HEG23	HAI	51.13	10.34	o 0 y H1	o 0 y H1	o 0 y H1	o i y H4/H10	o 0 y 0	o i y H10	o i y H10	o i y H10	o i y H10	o i y H12	o a y H12
HEG24	HAI	51.1	10.35	o 0 y H1	o 0 y H1	o 0 y H1	o i y H4/H10	o 0 y 0	o i y H10	o i y H10	o i y H10	o i y H10	o a y H12	o a y H12
HEG25	HAI	51.02	10.32	k 0 y 0	k 0 y 0	k 0 y 0	k 0 y H9	k 0 y H9	k 0 y H9	k 0 y H9	k 0 y H9	k 0 y H9	k 0 y H13	k 0 y 0
HEG26	HAI	51.28	10.37	k 0 y 0	o 0 y 0	o 0 y 0	o i y H4	o i y H4	o i y H4	o a y H4	o a y H4	o a y H4	o a y H12	o a y H12
HEG27	HAI	51.09	10.6	k 0 y 0	k 0 y 0	k 0 y 0	k 0 y H5	k 0 y H5	k 0 y H5	k 0 y H5	k 0 y H5	k 0 y H5	k 0 y H13	k 0 n 0
HEG28	HAI	51.27	10.5	k 0 y H2	k 0 n 0	k 0 n 0	k 0 n 0	k 0 n 0	k 0 n 0	k 0 y H2	k 0 y H4	k a y H4	o a y H12	o a y H12
HEG29	HAI	51.26	10.5	k 0 y H2	k 0 n 0	k 0 n 0	k 0 n 0	k 0 n 0	k 0 n 0	k 0 y H2	k 0 y H4	k a y H4	o a y H12	o a y H12
HEG3	HAI	51	10.43	k 0 n 0	k 0 n 0	k 0 n 0	k 0 n 0	k 0 n 0	k 0 n 0	k 0 n 0	k 0 n 0	k 0 n 0	k 0 n 0	k 0 n 0
HEG30	HAI	51.2	10.36	k 0 y H2	k 0 y H2	k 0 y H2	k 0 y H5	k 0 y H5	k 0 y H5	k 0 y H5	k 0 y H5	k 0 y H5	k 0 y H13	k 0 y H13
HEG31	HAI	51.17	10.22	k 0 y 0	k 0 y H4	k 0 y H4	k 0 y H5	k 0 y H5	k 0 y H5	k 0 y H5	k 0 y H5	k 0 y H5	k 0 y H15	k 0 y H15
HEG32	HAI	51.08	10.57	k 0 y 0	k 0 y 0	k 0 y 0	k 0 y H5	k 0 y H5	k 0 y H5	k 0 y H5	k 0 y H5	k 0 y H5	k 0 y H13	k 0 n 0
HEG33	HAI	51.11	10.43	k 0 y H2/H18	k 0 n 0	k 0 n 0	k 0 n 0	k 0 n 0	k 0 y 0	k 0 y H2	k 0 y H4	k a y H4	o a y H12	o a y H12
HEG34	HAI	51.21	10.39	o g y H1/H18	o g y H1/H18	o g y H1/H18	o g y H4	o g y 0	o g y H1/H18	o g y H1/H18	o g y H4	o g y H4	o g y H12	o g y H12
HEG35	HAI	51.22	10.41	o g y H1/H18	o g y H1/H18	o g y H1/H18	o g y H4	o g y 0	o g y H1/H18	o g y H4	o g y H4	o g y H4	o g y H12	o g y H12
HEG36	HAI	51.03	10.51	k 0 y H2	k 0 y H2	k 0 y H2	k 0 n 0	k 0 y 0	k 0 n 0	k 0 y 0	k 0 y 0	k 0 y 0	k 0 y H14	k 0 y H14
HEG37	HAI	51.03	10.51	k 0 y H2	k 0 y H2	k 0 y H2	k 0 n 0	k 0 y 0	k 0 n 0	k 0 y 0	k 0 y 0	k 0 y 0	k 0 y 0	k 0 y 0
HEG38	HAI	51.12	10.34	o 0 y H1	o 0 y H1	o 0 y H1	o 0 y H6	o 0 y 0	k 0 y H7	k 0 y H7	o 0 y H7	o 0 y H7	o a y H12	o a y H12
HEG39	HAI	51.12	10.35	o 0 y H1	o 0 y H1	o 0 y H1	o i y H4/H6	o 0 y 0	o i y H6	o i y H6	o i y H6	o i y H6	o i y H12	o i y H12
HEG4	HAI	51.11	10.44	k 0 y H2	k 0 n 0	k 0 n 0	k 0 n 0	k 0 n 0	k 0 n 0	k 0 n 0	k 0 n 0	k 0 n 0	k 0 n 0	k 0 y 0
HEG40	HAI	50.97	10.45	k 0 y 0	k 0 y 0	k 0 y 0	k 0 y H7	k 0 y H7	k 0 y H7	k 0 y H7	k 0 y H7	k 0 y H7	k 0 y H13	k 0 y H14
HEG41	HAI	51.22	10.37	k 0 y 0	k 0 y 0	k 0 y 0	k 0 y 0	k 0 y 0	k 0 y 0	k 0 y 0	k 0 y H9	k 0 y H8	k 0 y H16	k 0 y H16
HEG42	HAI	51.07	10.46	k 0 y 0	k 0 y 0	k 0 y 0	k 0 y H8	k 0 y 0	k 0 y H8	k 0 y H8	k 0 y H8	k 0 y H8	k 0 y H17	k 0 y H17
HEG43	HAI	51.3	10.44	k 0 y 0	k 0 y 0	k 0 y 0	k 0 y H6	k 0 y 0	k 0 y H8	k 0 y H8	k 0 y H8	k 0 y H8	k 0 y H16	k 0 y H16
HEG44	HAI	51.06	10.48	k 0 y H3	k 0 y H3	k 0 y H3	k 0 y H8	k 0 y H8	k 0 y H8	k 0 y H8	k 0 y H8	k 0 y H8	k 0 y H16	k 0 y H16
HEG45	HAI	51.04	10.51	k 0 y H3	k 0 y H3	k 0 y H3	k 0 n 0	k 0 y H11	k 0 y H8	k 0 y H8	k 0 y H8	k 0 y H8	k 0 y H16	k 0 y H16
HEG46	HAI	51.21	10.75	k 0 y 0	k 0 y 0	k 0 y 0	k 0 y H8	k 0 y H8	k 0 y H8	k 0 y H8	k 0 y H8	k 0 y H8	k 0 y H16	k 0 y H16
HEG47	HAI	51.28	10.37	k 0 y 0	k 0 y 0	o 0 y 0	o i y H4	o i y H4	o i y H4	o a y H4	o a y H4	o a y H4	o a y H12	o a y H12
HEG48	HAI	51.29	10.38	k 0 y 0	k 0 y 0	o 0 y 0	o i y H4	o i y H4	o i y H4	o a y H4	o a y H4	o a y H4	o a y H12	o a y H12
HEG49	HAI	51.28	10.39	k 0 y 0	k 0 y 0	o 0 y 0	o i y H4	o i y H4	o i y H4	o a y H4	o a y H4	o a y H4	o a y H12	o a y H12
HEG5	HAI	51.22	10.32	k 0 y H2	k 0 y H2	k 0 y H2	k 0 y H5	k 0 y H5	k 0 y H5	k 0 y H5	k 0 y H5	k 0 y H5	k 0 y H13	k 0 y H13
HEG50	HAI	51.28	10.42	k 0 y 0	k 0 y 0	k 0 y 0	o i y H4	o i y H4	o i y H4	o a y H4	o a y H4	o a y H4	o a y H12	o a y H12
HEG6	HAI	51.21	10.39	o g y H1/H18	o g y H1/H18	o g y H1/H18	o g y H4	o g y 0	o g y H4/H18	o g y H4	o g y H4	o g y H4	o g y H12	o g y H12
HEG7	HAI	51.27	10.41	k 0 y 0	o 0 y H4	o 0 y 0	o i y H4	o i y H4	o i y H4	o a y H4	o a y H4	o a y H4	o a y H12	o a y H12
HEG8	HAI	51.27	10.42	k 0 y 0	o 0 y H4	o 0 y 0	o i y H4	o i y H4	o i y H4	o a y H4	o a y H4	o a y H4	o a y H12	o a y H12
HEG9	HAI	51.22	10.38	o g y H1/H18	o g y H1/H18	o g y H1/H18	o g y H4	o g y 0	o g y H4/H18	o g y H4	o a y H4	o g y H4	k 0 y H16	k 0 y H16
SEG1	SCH	53.09	13.97	k 0 n 0	k 0 n 0	k 0 n 0	k 0 n 0	k 0 n 0	k 0 n 0	k 0 n 0	k 0 n 0	k 0 n 0	k 0 n 0	k 0 n 0
SEG10	SCH	53.11	14	k 0 n 0	k 0 n 0	k 0 n 0	k 0 n 0	k 0 n 0	k 0 n 0	k 0 n 0	k 0 n 0	k 0 n 0	k 0 n 0	k 0 n 0
SEG11	SCH	53.11	13.99	k 0 n 0	k 0 n 0	k 0 n 0	k 0 n 0	k 0 n 0	k 0 n 0	k 0 n 0	k 0 n 0	k 0 n 0	k 0 n 0	k 0 n 0
SEG12	SCH	53.09	13.97	k 0 n 0	k 0 n 0	k 0 n 0	k 0 n 0	k 0 n 0	k 0 n 0	k 0 n 0	k 0 n 0	k 0 n 0	k 0 n 0	k 0 n 0
SEG13	SCH	52.97	13.82	o a y S2	o a y S6/S2/S7	o a y S6/S2/S7	o a y S6/S7	o a y S6/S7	o a y S11/S8/S12/S9	o a y S11/S8/S12/S9	o a y S11/S8/S12/S9	o a y S11/S8/S12/S9	o a y S17	o a y S17
SEG14	SCH	53.09	13.98	k 0 n 0	k 0 n 0	k 0 n 0	k 0 n 0	k 0 n 0	k 0 n 0	k 0 n 0	k 0 n 0	o c y S12/S9	o c y S15/S17	o c y S15/S17
SEG15	SCH	53.11	14.01	k 0 y S3	k 0 y S3	k 0 y S3	k 0 n 0	k 0 n 0	k 0 n 0	k 0 n 0	k 0 n 0	k 0 y S10	k 0 y S15	k 0 n 0
SEG16	SCH	53.12	14	k 0 y S3	k 0 y S3	k 0 y S3	k 0 n 0	k 0 n 0	k 0 n 0	k 0 n 0	k 0 n 0	k 0 y S10	k 0 y S15	k 0 n 0
SEG17	SCH	53.1	13.63	o c y S14/S12	o c y S14/S12	o c y S14/S12	o c y S14/S12	o c y S14/S12	o c y S14/S12/S9	o c y S14/S12/S9	o c y S14/S12/S9	o c y S14/S12/S9	o c y S15/S17	o c y S15/S17/S1
SEG18	SCH	53.14	13.88	o a y S13/S5/S3	o a y 0	o a y 0	o a y S12	o a y S12	o a y S12/S9	o a y S18/S12/S9	o a y S18/S12/S9	o a y S18/S12/S9	o a y S15/S17	o a y S15/S17
SEG19	SCH	53.12	14.01	k 0 y S3	k 0 y S3	k 0 y S3	k 0 n 0	k 0 n 0	k 0 n 0	k 0 n 0	k 0 n 0	k 0 n 0	k 0 n 0	k 0 n 0
SEG2	SCH	53.09	13.98	k 0 n 0	k 0 n 0	k 0 n 0	k 0 n 0	k 0 n 0	k 0 n 0	k 0 n 0	k 0 n 0	k 0 n 0	k 0 n 0	k 0 n 0
SEG20	SCH	53.1	13.62	o c y S14/S12	o c y S14/S12	o c y S14/S12	o c y S14/S12	o c y S14/S12	o c y S12/S9	o c y S12/S9	o c y S12/S9	o c y S14/S12/S9	o c y S15/S17	o c y S15/S17/S1
SEG21	SCH	53.11	13.61	o c y S14/S12	o c y S14/S12	o c y S14/S12	o c y S14/S12	o c y S14/S12	o c y S12/S9	o c y S12/S9	o c y S12/S9	o c y S14/S12/S9	o c y S15/S17	o c y S15/S17/S1
SEG22	SCH	53.1	13.97	k 0 n 0	k 0 n 0	k 0 n 0	k 0 n 0	k 0 n 0	k 0 n 0	k 0 n 0	k 0 n 0	k 0 n 0	k 0 n 0	k 0 n 0
SEG23	SCH	53.11	14.03	k 0 y S3	k 0 y S3	k 0 y S3	k 0 n 0	k 0 n 0	k 0 n 0	k 0 n 0	k 0 n 0	k 0 y S10	k 0 n 0	k 0 n 0
SEG24	SCH	53.09	14	k 0 y S5/S3	k 0 y S3	k 0 y S3	k 0 y S3	k 0 y S3	k 0 y S10	k 0 y S10	k 0 y S10	k 0 y S10	k 0 n 0	k 0 n 0
SEG25	SCH	53.11	13.62	o c y S14/S12	o c y S14/S12	o c y S14/S12	o c y S14/S12	o c y S14/S12	o c y S12/S9	o c y S12/S9	o c y S12/S9	o c y S14/S12/S9	o c y S15/S17	o c y S15/S17/S1
SEG26	SCH	53.11	14.02	k 0 y S3	k 0 y S3	k 0 y S3	k 0 n 0	k 0 n 0	k 0 n 0	k 0 n 0	k 0 n 0	k 0 y S10	k 0 n 0	k 0 n 0
SEG27	SCH	53.12	13.71	o a y S14	o a y S12	o a y S12	o a y S12	o a y S12	o a y S12	o a y S12	o a y S12	o a y S12/S9	o a y S15/S17	o a y S15/S17
SEG28	SCH	53.09	14.01	k 0 y S3	k 0 y S3	k 0 y S3	k 0 y S3	k 0 y S3	k 0 y S10	k 0 y S10	k 0 y S10	k 0 y S10	k 0 n 0	k 0 n 0
SEG29	SCH	53.09	14	k 0 y S3	k 0 y S3	k 0 y S3	k 0 y S3	k 0 y S3	k 0 y S10	k 0 y S10	k 0 y S10	k 0 y S10	k 0 n 0	k 0 n 0
SEG3	SCH	53.1	13.99	k 0 n 0	k 0 n 0	k 0 n 0	k 0 n 0	k 0 n 0	k 0 n 0	k 0 n 0	k 0 n 0	k 0 n 0	k 0 n 0	k 0 n 0
SEG30	SCH	53.15	13.83	o a y S4/S3/S14	o a y S3/S14	o a y S12	o a y S12	o a y S12	o a y S12	o a y S12	o a y S12	o a y S12	o a y S15/S17	o a y S15/S17
SEG31	SCH	53.15	13.84	o a y S4/S3/S14	o a y S3/S14	o a y S12	o a y S12	o a y S12	o a y S12	o a y S12	o a y S12	o a y S12	o a y S15/S17	o a y S15/S17
SEG32	SCH	53.15	13.83	o a y S4/S3/S14	o a y S3/S14	o a y S12	o a y S12	o a y S12	o a y S12	o a y S12	o a y S12	o a y S12	o a y S15/S17	o a y S15/S17
SEG33	SCH	52.99	13.84	o a y S2	o a y S2/S7	o a y S2/S7	o a y S7	o a y S7	o a y S12/S9	o a y S12/S9	o a y S12/S9	o a y S12/S9	o a y S15/S17	o a y S15/S17
SEG34	SCH	52.98	13.85	o a y S2	o a y S2/S7	o a y S2/S7	o a y S7	o a y S7	o a y S12/S9	o a y S12/S9	o a y S12/S9	o a y S12/S9	o a y S15/S17	o a y S15/S17
SEG35	SCH	52.98	13.85	o a y S2	o a y S2/S7	o a y S2/S7	o a y S7	o a y S7	o a y S12/S9	o a y S12/S9	o a y S12/S9	o a y S12/S9	o a y S15/S17	o a y S15/S17
SEG36	SCH	52.99	13.84	o a y S2	o a y S2/S7	o a y S2/S7	o a y S7	o a y S7	o a y S12/S9	o a y S12/S9	o a y S12/S9	o a y S12/S9	o a y S15/S17	o a y S15/S17
SEG37	SCH	53.13	13.88	o a y 0	o a y 0	o a y 0	o a y S13	o a y S12	o a y S12	o a y S12	o a y S12	o a y S12	o a y S15/S17	o a y S15/S17
SEG38	SCH	53.12	13.68	o a y S14	o a y S12	o a y S12	o a y S12	o a y S12	o a y S12	o a y S12/S9	o a y S12	o a y S12/S9	o a y S15/S17	o a y S15/S17
SEG39	SCH	52.98	13.82	o a y S2	o a y S2/S7	o a y S2/S7	o a y S7	o a y S7	o a y S12/S9	o a y S12/S9	o a y S12/S9	o a y S12/S9	o a y S15/S17	o a y S15/S17
SEG4	SCH	53.11	14	k 0 y S3	k 0 y S3	k 0 y S3	k 0 n 0	k 0 n 0	k 0 n 0	k 0 n 0	k 0 n 0	k 0 y S10	k 0 y S15	k 0 n 0
SEG40	SCH	53.12	13.84	o a y 0	o a y 0	o a y 0	o a y S12	o a y S12	o a y S12	o a y S12	o a y S12	o a y S12	o a y S17	o a y S17
SEG41	SCH	53.12	13.85	o a y 0	o a y 0	o a y 0	o a y S12	o a y S12	o a y S12	o a y S12	o a y S12	o a y S12	o a y S17	o a y S17
SEG42	SCH	52.87	13.97	o h y S2	o h y S2/S12	o h y S2/S12	o h y S7	o h y S2/S12	o h y S2/S12	o h y S2/S12	o h y S2/S12	o h y S2/S12	o h y S15/S17	o h y S15/S17
SEG43	SCH	52.88	13.97	o h y S2	o h y S2/S12	o h y S2/S12	o h y S7	o h y S2/S12	o h y S2/S12	o h y S2/S12	o h y S2/S12	o h y S2/S12	o h y S15/S17	o h y S15/S17
SEG44	SCH	52.88	13.97	o h y S2	o h y S2/S12	o h y S2/S12	o h y S7	o h y S2/S12	o h y S2/S12	o h y S2/S12	o h y S2/S12	o h y S2/S12	o h y S15/S17	o h y S15/S17
SEG45	SCH	52.88	13.96	o h y S2	o h y S2/S12	o h y S2/S12	o h y S7	o h y S2/S12	o h y S2/S12	o h y S2/S12	o h y S2/S12	o h y S2/S12	o h y S15/S17	o h y S15/S17
SEG46	SCH	52.98	13.83	o a y S2	o a y S2/S7	o a y S2/S7	o a y S7	o a y S7	o a y S12/S9	o a y S12/S9	o a y S12/S9	o a y S12/S9	o a y S15/S17	o a y S15/S17
SEG47	SCH	52.99	13.83	o a y S2	o a y S2/S7	o a y S2/S7	o a y S7	o a y S7	o a y S12/S9	o a y S12/S9	o a y S12/S9	o a y S12/S9	o a y S15/S17	o a y S15/S17
SEG48	SCH	53.1	13.61	o c y S14/S12	o c y S14/S12	o c y S14/S12	o c y S14/S12	o c y S14/S12	o c y S12/S9	o c y S12/S9	o c y S12/S9	o c y S14/S12/S9	o c y S15/S17	o c y S15/S17/S1
SEG49	SCH	52.97	13.86	o a y S2	o a y S2/S7	o a y S2/S7	o a y S7	o a y S7	o a y S12/S9	o a y S12/S9	o a y S12/S9	o a y S12/S9	o a y S15/S17	o a y S15/S17
SEG5	SCH	53.11	14	k 0 y S3	k 0 y S3	k 0 y S3	k 0 n 0	k 0 n 0	k 0 n 0	k 0 n 0	k 0 n 0	k 0 y S10	k 0 y S15	k 0 n 0
SEG50	SCH	53.12	13.75	o c y S14	o c y S14	o c y S14	o c y S14	o c y S14	o c y S14	o c y S14	o c y S14	o c y S14	o c y S15/S17	o c y S15/S17
SEG6	SCH	53.1	13.62	o c y S14/S12	o c y S14/S12	o c y S14/S12	o c y S14/S12	o c y S14/S12	o c y S14/S12/S9	o c y S14/S12/S9	o c y S14/S12/S9	o c y S14/S12/S9	o c y S15/S17	o c y S15/S17/S1
SEG7	SCH	53.09	13.98	k 0 n 0	k 0 n 0	k 0 n 0	k 0 n 0	k 0 n 0	k 0 n 0	k 0 n 0	k 0 n 0	k 0 n 0	k 0 n 0	k 0 n 0
SEG8	SCH	53.11	14.02	k 0 y S3	k 0 y S3	k 0 y S3	k 0 n 0	k 0 n 0	k 0 n 0	k 0 n 0	k 0 n 0	k 0 y S10	k 0 y 0	k 0 n 0
SEG9	SCH	53.1	13.61	o c y S14/S12	o c y S14/S12	o c y S14/S12	o c y S14/S12	o c y S14/S12	o c y S14/S12/S9	o c y S14/S12/S9	o c y S14/S12/S9	o c y S14/S12/S9	o c y S15/S17	o c y S15/S17/S1

**Table 6.** Description of the agri-environmental measures coding of Table 5.

0 = no description or not applicable (0)
**Type of farming (1. digit)**
k = conventional
o = ecological
**Ecological directive (2. digit)**
a = EU-Bio
b = DE-Bio
c = Bioland
d = Naturland
e = Biokreis
f = Naturland
g = Demeter
h = Biopark
i = GÄA e.V.
**Agri-environmental measure (AEM) (3. digit)**
n = no
y = yes
**Description AEM (4.digit)**
**Alb**:
***MEKA (1992-2013/2014)***
A1 = N-B 1: extensive
A2 = N-B 2: extensive and low livestock density
A3 = N-B 3: steep slopes (inclination ≥ 25%) - difficult conditions
A4 = N-B 4: biodiverse
A5 = N-D 2: organic farming
A6 = N-G 1.1: extensive management of protected biotopes
***FAKT (2014-2020)***
A7 = B 1.1: extensive, max 1.4 RLU/ha, no mineral nitrogen
A8 = B 1.2: extensive, min 0.3 RLU/ha, no nitrogen
A9 = B 3: biodiverse, 4 indicator species
A10 = B 3.2: biodiverse, 6 indicator species
A11 = B 4: extensive management of protected biotopes
A12 = B 5: extensive management of FFH
A13 = C 1: meadow orchards
A14 = C 3: cattle breed
A15 = D 1: no chemical-synthetically plant protection agent or fertiliser
A16 = D 2.2: organic farming
A17 = N 6.1.1: NA (no information)
***Other***
A18 = SG 1: NA (no information)
A19 = landscape conservation guidelines: (unspecific, measures unknown)
A20 = forest biotope mapping: (unspecific, measures unknown)
**Hainich**
***KULAP (2000-2007)***
H1 = Programme A: unspecific (no information of subprogrammes)
H2 = Programme B: unspecific (no information of subprogrammes)
H3 = Programme C: unspecific (no information of subprogrammes)
***KULAP (2007-2014)***
H4 = L 1: organic farming
H5 = L 4: biodiverse
H6 = N 21: low-nutrient and dry habitats (grazing)
H7 = N 211: low-nutrient and dry habitats (cattle/horse grazing)
H8 = N 213: low-nutrient and dry habitats (sheep/goat grazing)
H9 = N 25: sheep farming and difficult terrain
H10 = N 311: low-nutrient and dry habitats (mowing)
H11 = N 312: low-nutrient and dry habitats (mowing, difficult conditions)
***KULAP (2014-2020)***
H12 = Ö2: organic farming
H13 = G 11: biodiverse
H14 = G21: biotope management by grazing
H15 = G31: biotope management by grazing and difficult conditions
H16 = G33: biotope management by sheep grazing
H17 = G53: biotope management by sheep grazing and difficult conditions
***Other***
H18 = FFH: NA (unspecific, measures unknown)
**Schorfheide**
***KULAP***
S1 = 33: disadvantaged sites (no further information)
S2 = 311A: extensive with no mineral fertiliser
S3 = 311C: extensive with no fertiliser
S4 = 313A: no mowing before 16 June
S5 = 313B: no mowing before 1 July
S6 = 413A: late and restricted management
S7 = 423B: late and restricted management
S8 = 613A: late and restricted management
S9 = 623B: organic farming
S10 = 661: extensive on farm level
S11 = 663: late and restricted management
S12 = 673: organic farming
S13 = 763: late and restricted management
S14 = 773: organic farming
S15 = 811A: extensive with no fertiliser
S16 = 812C: extensive management starts 1 July
S17 = 882: organic farming
***Other***
S18 = contractual nature conservation: Mowing after 15 June
